# Review of Incoherent Broadband Cavity-Enhanced Absorption Spectroscopy (IBBCEAS) for Gas Sensing

**DOI:** 10.3390/s18113646

**Published:** 2018-10-27

**Authors:** Kaiyuan Zheng, Chuantao Zheng, Yu Zhang, Yiding Wang, Frank K. Tittel

**Affiliations:** 1 State Key Laboratory of Integrated Optoelectronics, College of Electronic Science and Engineering, Jilin University, 2699 Qianjin Street, Changchun 130012, China; zhengkaiyuan1993@126.com (K.Z.); yuzhang@jlu.edu.cn (Y.Z.); ydwang@jlu.edu.cn (Y.W.); 2 Department of Electrical and Computer Engineering, Rice University, 6100 Main Street, Houston, TX 77005, USA; fkt@rice.edu

**Keywords:** incoherent broadband cavity-enhanced absorption spectroscopy (IBBCEAS), molecular spectroscopy, gas sensing, broadband light source, supercontinuum light source

## Abstract

Incoherent broadband cavity-enhanced absorption spectroscopy (IBBCEAS) is of importance for gas detection in environmental monitoring. This review summarizes the unique properties, development and recent progress of the IBBCEAS technique. Principle of IBBCEAS for gas sensing is described, and the development of IBBCEAS from the perspective of system structure is elaborated, including light source, cavity and detection scheme. Performances of the reported IBBCEAS sensor system in laboratory and field measurements are reported. Potential applications of this technique are discussed.

## 1. Introduction

Gas detection and precise quantitative concentration measurements are significant in many applications ranging from chemical analysis to atmospheric pollution monitoring [1,2,3]. Traditional methods for gas detection are based on the principle of chemical sensing. Samples are taken at a site and are then analyzed in the laboratory, resulting in unpredictable changes in sample quality and delays with respect to on-site processing. Absorption spectroscopy has become the foremost technique for the quantitative assessment of the concentration of atoms and molecules in the gas phase. Due to the advantages of high spatial and temporal resolution, real-time and in-situ measurement, absorption spectroscopy is widely used in the field of trace gas detection [4]. Different spectroscopic approaches for exploiting multiplexing features in a large spectral range have been reported, including cavity ring-down spectroscopy (CRDS) [5,6,7,8,9], cavity-enhanced absorption spectroscopy (CEAS) [10,11] and incoherent broadband cavity-enhanced absorption spectroscopy (IBBCEAS) [12]. These techniques offer different advantages and disadvantages in terms of selectivity, portability, sensitivity and cost.

Among these techniques for spectral absorption measurements, IBBCEAS technique is theoretically straight forward and capable of high sensitivity for trace gas detection. This technique was first reported in 2003 [12] and is based on the detection of the output light from a stable optical cavity with input light from an incoherent broadband light source. In IBBCEAS, a broadband light beam is firstly coupled into an optical cavity formed by two highly-reflective mirrors, and light leaked from the cavity is then dispersed with a grating monochromator and detected by a sensitive photodiode array or charge-coupled device (CCD) array. Concentration level of the gas species can be determined by a least-square algorithm that is commonly used in differential optical absorption spectroscopy (DOAS) [13,14].

The main advantages of IBBCEAS are as follows: (i) the experimental setup is simple, cost-effective and suitable for field measurements; (ii) no mode matching and mode-hop-free scanning are required, which differs from CEAS; (iii) IBBCEAS can be applied in the spectral range from ~190 nm (ultraviolet) to ~10 µm (infrared); (iv) multiple gas species can be measured simultaneously in a wide wavelength range. Therefore, IBBCEAS is widely applicable to both existing and new fields of gas sensing, chemical analysis and process control, water and air quality monitoring, remote vegetation sensing as well as explosive detection. This review mainly focuses on the application of IBBCEAS in gas sensing.

The structure of this review is organized as below. The first section covers the evolution of IBBCEAS and its main advantages compared with other techniques. The second chapter summarizes the principle of IBBCEAS for gas sensing. Section 3 describes the progress of IBBCEAS from the perspective of system structure. The fourth chapter focuses on the recent achievement of measurements of different gas species by IBBCEAS and highlights the obtained sensitivity and the minimum detection limit (MDL). Field measurements performances for different target gas species are demonstrated in the fifth section. In the last section, the main factors for performance improvement and the application prospects of the IBBCEAS technique are presented.

## 2. Principle of IBBCEAS for Gas Sensing

**Figure 1:**
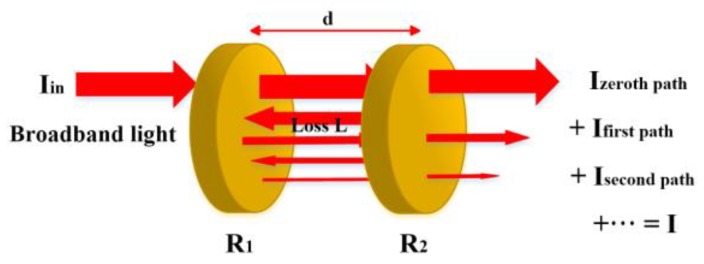
A principle diagram of light transmission in an optical cavity. The input of the broadband light *I*_in_ is coupled into an optical cavity with a length of d consists of two mirrors with a reflectivity of *R*_1_ and *R*_2_, respectively. Loss *L* represents the absorption by the target gas species. *I* is the total light intensity transmitted through the cavity.

In IBBCEAS, the time-integrated light intensity leaked from the cavity is inversely proportional to the absorption coefficient α of a specific gas molecule. The first application of an incoherent light source, a short-arc Xe lamp, was the measurement of the weak absorption of oxygen (O_2_) and of gaseous azulene (C_10_H_8_) [12]. A diagram of the light transmission in an optical cavity is shown in Figure 1. The optical cavity with a length of d consists of two mirrors with a reflectivity of *R*_1_ and *R*_2_, respectively. The input of the incoherent light intensity to the cavity is represented by Iin and the mirrors are assumed to absorb no light. Besides the transmission loss (1 − *R_i_*), resulting from the imperfect reflectivity of the mirrors, the main loss is the gas absorption (1 − *L*) after each light pass within the cavity. The total light intensity transmitted through the cavity can be expressed as the sum of each individual light intensity leaked from the cavity [12,15,16]. In this manner, the cavity output should be detected in wavelength scale by a device which is able to disperse or image the light. Therefore the output light can either be directly focused onto a monochromator with a CCD detector or be imaged onto an interferometer to measure the relation between the leaked light intensity from the cavity and the wavelength.

The cavity output can be written as

(1) $$I=I_{\mathrm{in}}\left(1-R_{1}\right)\left(1-L\right)\left(1-R_{2}\right)+I_{\mathrm{in}}\left(1-R_{1}\right)\left(1-L\right)R_{2}\left(1-L\right)R_{1}\left(1-L\right)\left(1-R_{2}\right)\;+\cdots +I_{\mathrm{in}}\left(1-R_{1}\right)\left(1-R_{2}\right)R_{1}^{n}R_{2}^{n}{\left(1-L\right)}^{2n+1}+\cdots +\;=I_{\mathrm{in}}\left(1-R_{1}\right)\left(1-R_{2}\right)\left(1-L\right)\overset{\infty }{\underset{n=0}{\sum }}R_{1}^{n}R_{2}^{n}{\left(1-L\right)}^{2n}$$

Since *R*_1_ < 1, *R*_2_ < 1 and *L* < 1, Equation (1) can be written as

(2) $$I=I_{\mathrm{in}}\frac{\left(1-R_{1})(1-R_{2})(1-L\right)}{1-R_{1}R_{2}{\left(1-L\right)}^{2}}$$

For a cavity without absorption loss, i.e., *L* = 0, Equation (2) can be simplified to

(3) $$I=I_{\mathrm{in}}\frac{\left(1-R_{1}\right)\left(1-R_{2}\right)}{1-R_{1}R_{2}}$$

Consequently, the single-pass loss can be expressed as the difference between the light intensity measured in a cavity filled with a target gas (*I*) and that without the gas (*I*_0_)

(4) $$1-L=\pm \sqrt{\frac{1}{4}{\left(\frac{I_{0}}{I}\frac{1-R_{1}R_{2}}{R_{1}R_{2}}\right)}^{2}+\frac{1}{R_{1}R_{2}}}-\frac{1}{2}\frac{I_{0}}{I}\frac{1-R_{1}R_{2}}{R_{1}R_{2}}$$

Assuming that the single-path loss is related to Lambert-Beer law as 1−L=exp(−αd) and *R*_1_ = *R*_2_ = *R*, the absorption coefficient α can be written in the following form:

(5) $$\alpha =\frac{1}{d}\left|\ln\left(\frac{1}{2R^{2}}\left(\sqrt{4R^{2}+{\left(\frac{I_{0}}{I}\left(R^{2}-1\right)\right)}^{2}}+\frac{I_{0}}{I}\left(R^{2}-1\right)\right)\right)\right|$$

It should be noted that the derivation of Equation (5) does not use any approximation related to *α* and *R*, and thus even in the condition of a large *α* and a small *R*, Equation (5) is applicable. In case of a small loss per pass (*L* → 0) and a high reflectivity of the mirrors (*R* → 1), *α* can be approximated by:

(6) $$\alpha \approx \frac{1}{d}\left(\frac{I_{0}}{I}-1\right)\left(1-R\right)$$

Equation (6) indicates that the effective path length in IBBCEAS can be increased by a factor of ${\left(1-R\right)}^{-1}$ in comparison to single-pass. The effective absorption path length is ${\left(1-\sqrt{R_{1}R_{2}}\right)}^{-1}$ times that of a single-pass in case of a general condition of *R*_1_ ≠ *R*_2_. Furthermore, the signal-to-noise ratio (SNR) is increased by a factor of ${\left[2\left(1-R\right)\right]}^{-1/2}$ [17]. Fiedler et al. [12] studied the impact of mirror reflectivity, cavity length and light injection pattern on the cavity output [18], which clearly demonstrated the relationship between these parameters.

**Figure 2:**
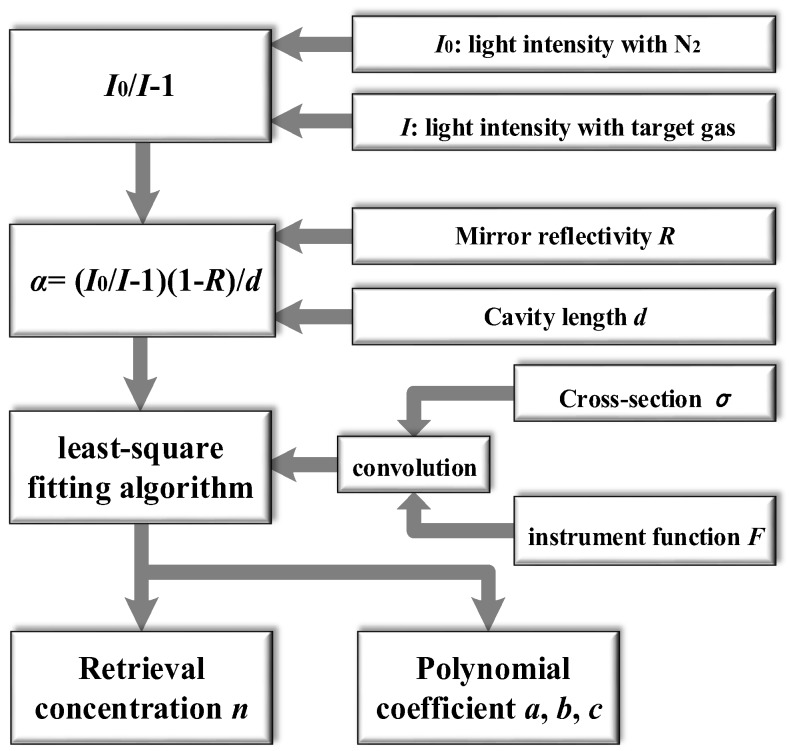
Concentration retrieval procedure for IBBCEAS.

Given the measured mirror reflectivity *R* and cavity length *d*, the target gas concentration can be retrieved by a least-square fitting algorithm [19]. As shown in Figure 2, the main concept of this method is fitting the cross-section *σ* of the target gas to the experimentally measured absorption coefficient *α* as [9]

(7) $$\alpha =\underset{i}{\sum }n_{i}\cdot \sigma_{i}+a\lambda^{2}+b\lambda +c$$

where $\sigma_{i}$ represents the reference cross-section of the *i*th gas species, which should be convoluted with the measurement instrument function and can be obtained from the HITRAN database, $n_{i}$ is the concentration of the *i*th gas species. The second-order polynomial $a\lambda^{2}+b\lambda +c$ represents the background baseline, which could arise from the light source intensity and cavity coupling variations over the time, mechanical vibration, gas scattering including Rayleigh and Mie scattering, aerosol extinction and system drifts. This fitting method is widely used in DOAS for spectral fitting, which is rather effective in retrieving concentrations even in cases where the spectrum appears noisy and shows baseline drifts. The unknown parameters (*n_i_*, *a*, *b* and *c*) can be extracted using a linear algebraic method such as the singular value decomposition (SVD) method or nonlinear Levenberg-Marquardt routine for real-time concentration retrieval. Temperature and pressure mainly affect the shape and profile of the absorption line. When we convolute the instrument function with the reference cross-section, temperature and pressure broadening effect should be taken into account.

## 3. Experimental Aspects

**Figure 3:**
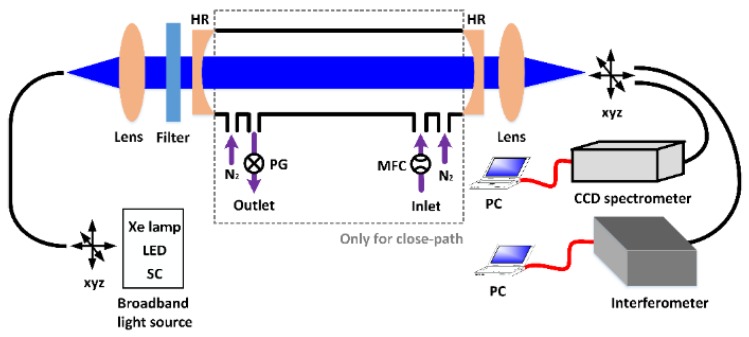
Schematic diagram of a typical IBBCEAS setup. HR is a high-reflectivity mirror used to form an optical cavity. CCD spectrometer is a charge-coupled device spectrometer. PC is a personal computer for data processing. PG represents the pressure gauge and MFC is the mass flow controller. The dashed box refers to the structure of the close-path cavity, which is unnecessary for an open-path configuration.

Figure 3 shows a schematic diagram of a general IBBCEAS set-up. A broadband light source can be a short-arc Xe lamp, a light emitting diode (LED) or a supercontinuum (SC) source. The incoherent light is coupled directly from the source into an optical fiber. The emerging light from the fiber was collimated with a lens and then injected into a high-finesse optical cavity. A filter was located in front of the cavity, in order to remove the wavelength components outside the highly reflective range. The optical cavity is formed by two reflective dielectric mirrors separated by a specific distance. The beam in the cavity is reflected between the two mirrors, resulting in an improvement of the effective optical path length. For a close-path configuration, the sample flow is controlled by means of a mass flow controller at the inlet of the cavity. A pump and a pressure gauge are used at the outlet to pump the target gas into the cell and to control the gas pressure inside the cavity, respectively. In addition, purified gas (such as N_2_) is introduced adjacent to both mirrors in order to prevent mirror pollution by aerosol deposition and volatile organic compounds (VOCs). For an open-path configuration, none of these devices described above is needed. Light transmitted through the cavity is focused with a lens and coupled into an optical fiber. The fiber output can be directly connected to a CCD spectrometer or imaged onto an interferometer. The length of the cavity and the radius of curvature of the mirror determine the volume of the chamber.

Fang et al. [20] reported a novel IBBCEAS instrument utilizing a custom cage optical system with a size of 676 mm × 74 mm × 86 mm and a weight of 4.5 kg. The sensor system for transmitter (LED output) and receiver units (CCD spectrometer) were interchangeable, which realized a compact and stable optical system that could be easily aligned. In the following Section 3.1, Section 3.2 and Section 3.3, we will describe the development of IBBCEAS from the perspective of system structure, including light source, cavity and detection scheme.

Table 1 shows the important items (light source, cavity and detection scheme) used in IBBCEAS and the corresponding advantages and disadvantages. With respect to long-term test and gas sensing in field application, the system configuration combining an LED with a CCD spectrometer maybe more suitable since they are more cost-effective, faster as well as more portable for mobile deployment. For high-resolution spectral measurements, the SC source based Fourier-transform spectrometer (FTS) is more preferable due to the ultrahigh-resolution of the FTS with a high brightness of the SC source.

### 3.1. Light Source

**Figure 4:**
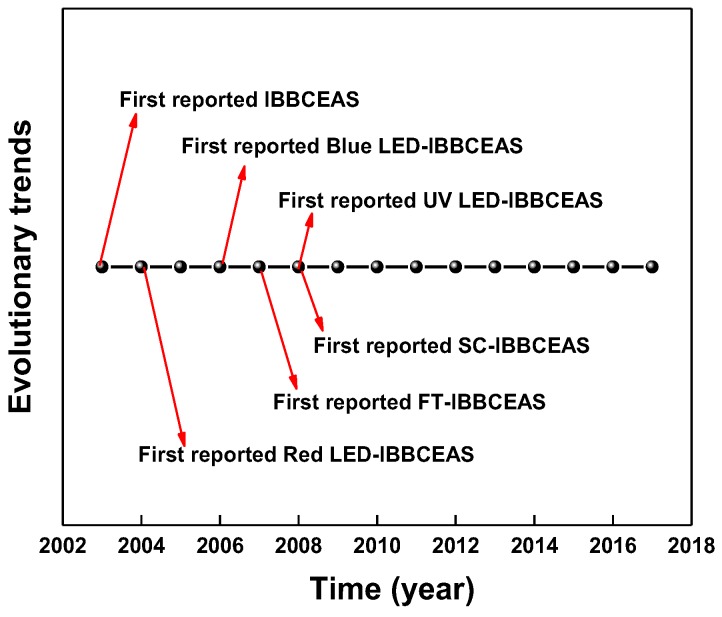
Time scale where different types of sources were firstly used to implement IBBCEAS since 2003.

The choice of a broadband light source depends crucially on its application and also impinges on the SNR of a sensor system. Generally, the favorable attributes of a light source should include robustness, low cost, long lifetime, and high photon fluence with enough SNR to allow for adequate coupling efficiency of the broadband light to a high-finesse cavity. In addition, it should have a wide emission spectrum in the wavelength range of interest for the purpose of a high degree of flexibility in terms of switching among different excitation ranges. Also, a high emission stability and a low spectral drift are of importance for achieving a good sensor performance. However, no broadband light source meets all these requirements and thus some of these properties will be a compromise. Figure 4 shows the scale of time where different type of light sources was adopted to implement IBBCEAS. This is followed by a summary of the main characteristics of light sources commonly used in IBBCEAS.

#### 3.1.1. Short-Arc Xe Lamp

An arc lamp emits white light covering a wide spectral region from the ultraviolet (UV) to the near-infrared (near-IR) [21]. Due to the high spectral brightness and low intensity fluctuations, Xe lamp-based IBBCEAS was firstly demonstrated in 2003 [12]. A short-arc Xe lamp with a specified luminosity of 18 W·cm^−2^·sr^−1^·nm^−1^ at 400 nm with specific optical filter and fluctuations of up to ~10% was successfully employed. Since then, this kind of light source has been used in numerous applications using IBBCEAS [9,12,22,23,24,25,26,27,28,29,30,31,32]. Varma et al. [9] placed a 75 W Xe lamp in a water-cooled housing with an F/2 ellipsoidal reflector in order to stabilize the light source and improve the light collection efficiency. High-resolution applications in IBBCEAS usually require a Fourier transform spectrometer. By adopting a small-volume chamber, Orphal et al. [23] reported the first application of the combination of Fourier transform spectroscopy (FTS) and IBBCEAS in the near-IR band ranging from 5800 cm^−1^ to 7000 cm^−1^. Utilizing a long averaging time, high-frequency noise introduced by the lamp fluctuation was avoided.

#### 3.1.2. LED

LEDs, which are widely used nowadays in multi-media and lighting, are available for the spectral region from the near-UV to the near-IR. And such a wide spectral range allow access to almost all molecules exhibiting strong absorption arising from fundamental vibrational–rotational transitions with a specific molecular signature [33]. Due to high brightness, low power consumption, narrow full width at half maximum (FWHM) and long life time, LEDs are suitable for IBBCEAS applications in the sensitive detection of numerous important atmospheric trace gas species and pollutant gases. The first application of LEDs for IBBCEAS was demonstrated by Ball et al. [34] used red and green LEDs with different optical filters. Since then, LED-based IBBCEAS have been widely applied in IBBCEAS for multiple trace gas detection ranging from near-UV to the visible (VIS) band. Furthermore, it should be noted that LEDs are sensitive to temperature fluctuations and hence adequate temperature and current stabilization in gas detection are required.

Superluminescent LEDs (SLEDs) have shown to be a good choice for IR gas sensing since their emission wavelengths correspond to the high reflectivity of the cavity mirrors. They are based on the generation and amplification of spontaneous emission in a semiconductor waveguide. In addition, a SLED has a relatively high spectral power density and a high spatial coherence in the near-IR range. However, SLEDs have not been widely adopted as light sources for IBBCEAS measurements to-date [35,36].

#### 3.1.3. SC Source

SC radiation sources are attractive and broadly used in spectroscopic applications, such as in physics, chemistry and biology [37,38,39,40], owing to the combination of high power density and wideband wavelength coverage [41]. A supercontinuum excitation source requires a seed laser with a high repetition rate that determines the SC mode spacing and an achromatic lens to collimate the SC radiation. Light exits the photonic crystal fiber (PCF) into a beam shows a high power density and an ultra-wide spectral region access.

The application of SC light source for IBBCEAS was first demonstrated by Langridge et al. [42] for the quantitative measurements of NO_2_ and NO_3_. Other reports of SC sources applied in IBBCEAS are within the VIS to the near-IR regions. An interesting approach to create continuum radiation for IBBCEAS was presented by Ruth et al. [43], by using a short-pulse laser to stimulate a high-temperature black body inside the cavity. This method generates SC radiation directly in the cavity instead of using a PCF, resulting in a consecutive emission of the light and notable enhancement of coupling efficiency.

### 3.2. Cavity Scheme

The most common reported geometry of a cavity for gas sensing is the conventional linear mirror cavity formed by two highly reflective mirrors separated by a specific distance. The target gas can be continuously pumped from a sample volume and injected into the cavity through a closed-path cavity configuration. Alternatively, some atmospheric monitoring applications use an open-path configuration, especially for in-situ field measurements together with an atmospheric simulation chamber. Both closed-path and open-path configurations present advantages and drawbacks for trace gas sensing.

#### 3.2.1. Closed-Path Configuration

Zhao et al. demonstrated. a closed-path configuration with a typical cavity length of ~1 m [19], which enables a portable broadband sensor system with good mechanical stability and compactness. A major advantage of a closed cavity is that the calibration of the mirror reflectivity can be measured by filling the cavity with a calibration gas (for CEAS applications) or zero air (for CRD applications). Furthermore, optical loss of aerosol can be eliminated by filtering the pumped gas sample on the inlet side. Teflon polytetrafluoroethylene (PTFE) filter was adopted by a majority of reported systems to remove ambient aerosols [19,44,45].

The main drawback of the closed-path system is related to the wall losses for different target gas species [46]. They occur on the inlet side and on the wall of the enclosed cavity over time, especially for some “sticky” gases, such as NO_2_ and NH_3_ etc. Most of the reported chambers are made of Perfluoroalkoxy alkanes (PFA) to minimize gas loss during detection. For the sake of mirror protection, a small purge flow of zero air or N_2_ is usually used on both sides of the cavity.

#### 3.2.2. Open-Path Configuration

Unlike an enclosed cavity, an instrument using an open-path cavity is free from wall losses of the target gas species, as reported by Wu et al. [47]. Since the cavity mirrors are not fixed, the cavity length can be extended arbitrarily, which improves the sensor sensitivity. Moreover, a long cavity enables the use of mirrors with a low reflectivity without decreasing the SNR of the cavity transmission and the retrieval accuracy of the target gas species.

However, for high aerosol concentration levels, the concentration retrieval of the target gas species may be challenging, owing to the significant reduction of the effective optical path length for open-path configurations. Moreover, another drawback of open-path configuration is the unavailability of a “clean air” reference spectrum *I*_0_, which is problematic for mirror reflectivity calibration and concentration measurements. But in simulation chambers [9,25,31] with open-path configuration, this issue can be solved in the way of filling the chamber with a calibration gas or zero-air.

### 3.3. Detection Scheme

In terms of detection scheme, the light transmitted through the cavity can either be directly focused onto the entrance aperture of a monochromator equipped with a CCD detector or be imaged onto the entrance aperture of an interferometer. Pros and cons of the two methods will be presented in Section 3.3.

#### 3.3.1. Dispersive Approach

The light leaking out of the cavity is either coupled into a fiber attached to a monochromator or directly focused onto the entrance slit of a monochromator. Dispersive optical elements, typically a reflective grating, usually choose a single order and are then imaged onto a CCD array. The spectral resolution is usually determined by the monochromator’s slit width, the groove density of the grating and the distance between the injection point and the CCD detector in conjunction with its pixel size. Spectral resolutions between ~0.1 nm and ~1 nm are sufficient to resolve broadened spectral features in the UV–VIS range [9,10]. Due to a high response speed of the CCD, this method processes a merit of rapid detection over a wide spectral range but is not able to perform high-resolution spectroscopic detection.

#### 3.3.2. Interferometric Approach

The light transmitted through the cavity is imaged into an interferometer, and is then separated by a beam splitter. A continuously moving mirror shifts back and forth along the optical axis, making the light travel over different path lengths inside the interferometer. The optical path difference between the two light beams is periodically changed and interference occurs. The interference signal is converted into an electrical signal by a single-channel detector, and a digital interferogram is obtained by means of an analog-to-digital (A/D) converter. Finally, the spectrum information is reconstructed after performing the Fourier Transform on the recorded interferogram. The spectral resolution is determined by the size of the aperture and the maximum path length difference caused by the two interfering beams. The typical resolution of IBBCEAS is between 0.02 cm^−1^ and 4 cm^−1^ from the VIS to the near-IR range. However, high resolution and sensitivity come at the expense of rather long acquisitions times. But such approaches are suitable for chemical flow reactors that require small sample volume, and can accurately measure the spectrum of a gas molecule.

Fourier-transform IBBCEAS (FT-IBBCEAS) was first demonstrated by Ruth et al. [22] by using a commercial FTS in a standard IBBCEAS setup using short-arc Xe lamp as light source. The spin-forbidden B-band of gaseous oxygen at 688 nm as well as the weak absorption transitions of water vapor was measured. The absorption spectra of the overtone bands of CO_2_, OCS and HD^18^O in the near-IR were also detected in further FT-IBBCEAS studies [23].

## 4. Target Gas Species Performances in Laboratory

**Figure 5:**
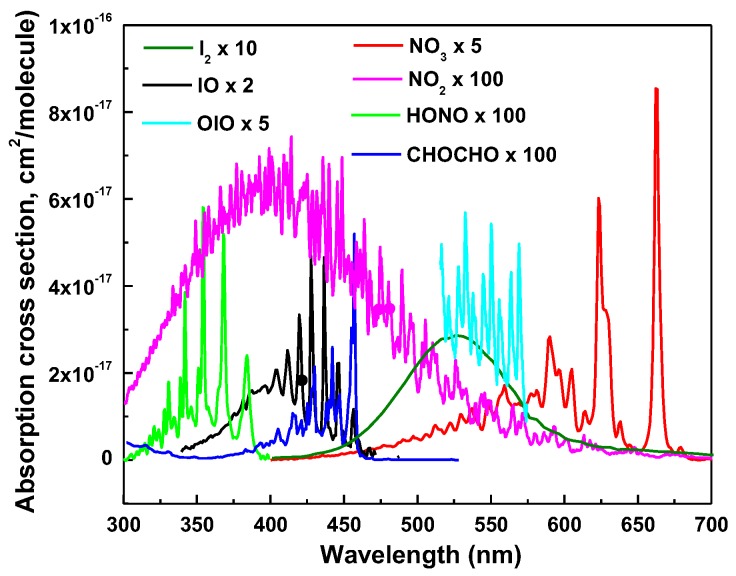
Spectra of key atmospheric molecular species in the UV and VIS region (×10 means the cross section is multiplied by ten times, and so on).

There is a substantial increment in the quantity of literature reporting IBBCEAS and its variants techniques over the past decade. IBBCEAS has been successfully applied in atmospheric and environmental measurements [48,49]. Trace gas species are usually involved in the atmospheric chemical cycle and have a residence time in the atmosphere of a few days to several decades or even longer. Some gas species are naturally generated by human activities. They are subject to physical, chemical, biological earth processes and are involved in biogeochemistry circulation, such as NO_3_, NO_2_, I_2_, CHOCHO and CO_2_. This section attempts to review the latest research and development of trace gas detection in laboratory using the IBBCEAS technique, including proof-of-principle of this technique, multiple and single target gas detection. Spectra of key atmospheric molecular species in the UV and VIS region are depicted in Figure 5. In Table 2, it shows the performances of the reported IBBCEAS instrument for key gas species detection.

### 4.1. Proof-of-Principle of the IBBCEAS (O_2_, C_10_H_8_, CO_2_, OCS, HD^18^O)

The proof-of-principle of the IBBCEAS technique can be demonstrated by measuring the weak atmospheric transition of various materials, such as the $b^{1}\overset{+}{\underset{g}{\sum }}\left(v^{\prime }=2\right)\leftarrow X^{3}\overset{-}{\underset{g}{\sum }}\left(v^{\prime \prime }=0\right)$ absorption transition of O_2_ and the $S_{1,v^{\prime }}\leftarrow S_{0}$ absorption spectrum of azulene at its vapour pressure at room temperature. In addition, absorption spectra in the overtone bands of CO_2_, OCS and HD^18^O were also measured using the IBBCEAS technique.

In 2003, Fiedler et al. [12] demonstrated a new highly-sensitive IBBCEAS using a short-arc Xe lamp with a 45 cm-long cavity, and the output light was detected by a grating monochromator equipped with a photodiode array. The absorption spectrum of the $b\left(v^{\prime }=2\right)\leftarrow X\left(v^{\prime \prime }=0\right)$ transition (Q/R and part of P branch) of O_2_ at 1000 mbar in a static cell between 628 nm and 630 nm was measured with IBBCEAS at a 25 s averaging time, together with the $S_{1}\leftarrow S_{0}$ absorption spectrum of gaseous azulene at an averaging time of 90 s, resulting in an absorption coefficient of 1.3 × 10^−5^ cm^−1^ within the 628 nm and 670 nm spectral range.

For high resolution applications requiring Fourier transform detection, a new design of IBBCEAS was proposed in 2007. Ruth et al. [22] adopted a commercial FTS in a standard IBBCEAS set-up in place of a monochromator using a short-arc Xe lamp. This new technique, named FT-IBBCEAS, was applied for measuring the transition of $b^{1}\overset{+}{\underset{g}{\sum }}\left(v^{\prime }=1\right)\leftarrow X^{3}\overset{+}{\underset{g}{\sum }}\left(v^{\prime \prime }=0\right)$ of O_2_ at ~688 nm and several transitions of H_2_O in ambient air at ~720 nm, leading to a path-length enhancement factor of 200 and a SNR improvement of ~6 compared to the single-pass absorption measurement. In 2008, the same group [23] extended the spectral region to the near-IR, and the absorption spectra of the overtone bands of CO_2_, OCS, and HD^18^O were measured between 5800 cm^−1^ and 7000 cm^−1^ with a data acquisition time of 90 min. Furthermore, it was demonstrated that the high-resolution spectra provided an improved method to calibrate mirror reflectivity or an absolute absorption cross-section without measuring an empty cavity using a small sampling volume, but at the expense of time resolution.

A new method in the application of IBBCEAS for measuring the absorption spectrum of C_10_H_8_ as well as the $b^{1}\overset{+}{\underset{g}{\sum }}\left(v^{\prime }=2\right)\leftarrow X^{3}\overset{-}{\underset{g}{\sum }}\left(v^{\prime \prime }=0\right)$ of O_2_ was demonstrated by Ruth et al. [43] in 2015. The main idea was to place a laser-induced gas plasma, generated by a short pulse laser, in the center of a quasi-confocal high-finesse cavity, which was used as the pulsed incoherent broadband light source. The light emission was sustained by the cavity despite the initially large optical loss. Experiments demonstrated a good agreement (*R*^2^ = 0.9985) in measuring part of the absorption spectrum of C_10_H_8_ and the strongly forbidden *γ*-band in O_2_ compared with the CRDS in the same region [66].

### 4.2. Multiple Target Gas Detection

#### 4.2.1. Simultaneous Detection of NO_3_ and NO_2_

Reliable concentration assessment of the major atmospheric oxidants and their precursors is essential to advance our understanding of chemical process in the troposphere [25,67,68,69,70,71]. Most reported IBBCEAS set-ups for simultaneous detection of NO_3_ and NO_2_ are in a spectral range of 610−690 nm using Xe lamps and LEDs.

In 2006, Venables et al. [25] used a 75 W Xe lamp source and a 462 cm atmospheric simulation chamber with two mirrors (*R*~99.775%). The cavity output was directed into a spectrograph equipped with a CCD detector. A sensitivity of 4 pptv for NO_3_ and 10 ppbv for NO_2_ was obtained between 620 nm and 690 nm in a 1 min acquisition time. In 2009, Varma et al. [9] used a 20 m-long optical cavity to detect NO_3_ and NO_2_ between 630 nm and 690 nm. The reported MDL, defined as the minimum concentration level that can be detected by the sensor, was experimentally determined to be ~2 pptv for NO_3_ and 2 parts-per-billion in volume (ppbv) for NO_2_ with an acquisition time of 5 s. In 2010, a progress in cavity length stated to be 50 cm was proposed by Triki et al. [50]. Furthermore, they also improved their set-up reported before [57] by connecting an optical fiber to a spectrometer, which degraded the spectrometer resolution from ~1.8 nm to ~2.3 nm but dramatically increased the light intensity on the CCD array. A MDL of 2 pptv for NO_3_ and 600 pptv for NO_2_ were achieved with a ~400 s averaging time. In 2014, Wu et al. [51] proposed an experiment using an atmospheric chamber (8 m^3^) using a cavity with mirrors of a reflectivity of 99.991% between 638 nm and 672 nm, leading to a maximum effective optical path length of 22 km. In this paper, an IBBCEAS instrument was used in a simulation chamber to monitor NO_3_, NO_2_ concentration levels in chemical reaction. The reported MDL for NO_3_ and NO_2_ were 7.9 pptv and 9.0 ppbv respectively with an acquisition time of 60 s.

#### 4.2.2. Simultaneous Detection of NO_3_ and N_2_O_5_

For simultaneous detection of NO_3_ and N_2_O_5_, most IBBCEAS devices are within 615−706 nm using red LEDs. A novel application in IBBCEAS instrumentation was reported by Kennedy et al. [52] with the idea of airborne measurements in night time using three channels (i.e., three cavities) in an IBBCEAS instrument. The advancement in this paper was to establish a new clever way in which the mirror reflectivity can be determined. Channel one and two are used for NO_3_ and N_2_O_5_ detection with two red LEDs centered at 685 nm. The 1*σ* standard deviation indicated a MDL for N_2_O_5_ of 2.4 pptv and for NO_3_ of 1.1 pptv, with an uncertainty of 14% and 11%, respectively. In 2017, Wang et al. [44] demonstrated a set-up based on a 665-nm LED with a 50 cm-long cavity formed by two mirrors (*R*~99.99%). This work focused on the simultaneous detection of NO_3_ and N_2_O_5_. The instrument reported a novel design of mechanically aligned non-adjustable optical mounting system, which enabled a fast set-up and a stable operation in field applications for trace gas detection. That instrument allowed a MDL of 2.4 pptv (1*σ*) and 2.7 pptv (1*σ*) for NO_3_ and N_2_O_5_, respectively. In a 1 s interval, the associated uncertainties were estimated to be 19% for NO_3_ and 22−36% for N_2_O_5_, respectively.

#### 4.2.3. Simultaneous Detection of HONO and NO_2_

A spectral range of 353–387 nm is communicated for most IBBCEAS set-ups for simultaneous detection of HONO and NO_2_. In 2008, Gherman et al. [53] reported the first application of IBBCEAS in the near-UV for the simultaneous detection of HONO and NO_2_. The obtained MDL was ~4 ppbv for HONO and ~14 ppbv for NO_2_ in a 20 s acquisition time using a static gas cell set-up with two mirrors (*R* ~ 99.85%) separated by distance of 1.15 m. Moreover, an atmospheric simulation chamber (4 m^3^) with a cavity length of 4.5 m was also proposed in this paper. Minimum detection limits of ~0.13 ppbv for HONO and ~0.38 ppbv for NO_2_ were achieved in a 10 min acquisition time. An open-path detection allows one to avoid absorption cell wall losses and sampling induced artifacts. In 2012, Wu et al. [47] demonstrated an experiment for open-path measurements of HONO and NO_2_ using a UV LED centered at 365 nm. The cavity length was ~1.85 m and minimum detection limits were given as ~430 pptv for HONO and ~1 ppbv for NO_2_ in 90 s.

With respect to the IBBCEAS for field application, in 2014, Wu et al. [54], for the first time, demonstrated the feasibility of the simultaneous measurement of ambient HONO and NO_2_ concentration levels in a field campaign that are free from chemical and spectral interferences at a suburban site of Tung Chung in Hong Kong. A MDL (2*σ*) of 0.6 ppbv for HONO and 2 ppbv for NO_2_ were achieved with an optimum acquisition time of 120 s. In 2015, Duan et al. [55] reported an IBBCEAS for field measurements in Anhui, China, which was able to detect a HONO concentration level of 0.22 ppbv and NO_2_ of 0.45 ppbv (1*σ*) with an acquisition time of 320 s. In order to determine the precision of the IBBCEAS sensor, concentration levels of HONO and NO_2_ were recorded for three days, and the results agreed well (*R*^2^ = 0.917) with those obtained by a commercial DOAS device.

#### 4.2.4. Simultaneous Detection of CHOCHO and NO_2_

Glyoxal (CHOCHO) is the simplest alpha-dicarbonyl and is present in the atmosphere as a first generation product from VOCs [20] and leads to the formation of secondary organic aerosol (SOA) and ozone (O_3_) [72,73,74]. Although glyoxal is important in SOA formation and photochemistry, until recently there have been only a few techniques for rapid and in-situ atmospheric glyoxal measurements.

In 2008, Washenfelder et al. [27] reported the first simultaneous measurements of CHOCHO and NO_2_ by IBBCEAS. Broadband light generated by a Xe arc lamp was coupled into an optical cavity consisting of two highly-reflective mirrors (*R*~99.9966%) with a separation distance of 94.4 cm. For a 1 min integration time, the measurement precision (±1*σ*) for CHOCHO and NO_2_ were 29 pptv and 20 pptv, respectively. Another important step towards measuring CHOCHO and NO_2_ was taken by Fang et al. [20], and a special IBBCEAS instrument was developed by utilizing a custom cage system and a 42 cm-long cavity. This set-up was used for sensitive, real-time, in-situ measurement of CHOCHO and NO_2_ in the spectral range of 440−480 nm. A measurement precision of CHOCHO and NO_2_ in ambient air was reported to be 28 and 50 pptv at a 60 s averaging time, and the accuracy was reported to be 5% and 4% for the two gas species, respectively. Furthermore, a Kalman adaptive filter method was applied to retrieve gas concentration, leading to a precision improvement of CHOCHO and NO_2_ to 8 pptv and 40 pptv in a 21 s averaging time.

#### 4.2.5. Simultaneous Detection of I_2_, IO and OIO

Iodine is an important trace gas species in the earth’s atmosphere [75,76,77,78,79]. It can affect atmospheric oxidation capacity in a number of ways, including the catalytic destruction of O_3_, generating iodine oxides such as IO and OIO and also can lead to the formation of marine aerosol [80,81,82,83,84,85,86]. In 2008, Vaughan et al. [26] reported a novel combination of IBBCEAS with a discharge-flow tube for the study of I_2_, IO and OIO. Light from a 150 W short-arc Xe lamp was focused into a 145 cm-long cavity, leading to a MDL of ~26 pptv for I_2_ in a fitting range between 525 nm and 555 nm, indicating an effective optical path length of 81 cm in a 60 s acquisition time. A MDL of ~45 pptv for OIO was achieved in the same fitting range with an effective optical path length of 42.5 cm in a 5 s acquisition time. Moreover, a MDL of ~210 pptv for IO was obtained between 420 nm and 460 nm in 60 s. In 2012, Ashu-Ayem et al. [32] investigated the time profile of I_2_ emissions. In this experiment, the algal specimens were exposed to an atmosphere simulation chamber under controlled conditions. A 75 W xenon arc lamp and two individual channels including a blue one (420–460 nm) for IO measurement and a green one (520–560 nm) for I_2_ and OIO detection were used. Two mirrors (*R*~99.97%) with a distance of 199 cm were adopted to form a cavity. The results showed a good agreement between the fitted spectra and the measured one, which indicated that all significant spectral features can be extracted from the corresponding fitting range. The emission rate varied from 7 pmol·min^−1^·gFW^−1^ to 616 pmol·min^−1^·gFW^−1^ in O_3_-free air with an average value of 55 pmol·min^−1^ gFW^−1^.

### 4.3. Single Target Gas Detection

#### 4.3.1. NO_3_

For NO_3_ measurement, most IBBCEAS devices are within 640–690 nm using red LEDs. Ball et al. [34] demonstrated the first use of red LED in IBBCEAS, owing to the primary advantage of low-cost, robustness and energy efficiency of the LED [87]. In this paper, the red LED light was coupled into a 1.9 m-long optical cavity for NO_3_ detection. Concentration level of 40.3 ± 2.5 pptv in ambient air was reported in 516 s, while the root mean square (RMS) of the residual spectrum was stated to be 5.9 × 10^−9^ cm^−1^. In 2008, Langridge et al. [56] reported an instrument for the quantitative measurements of NO_3_. The reported 1*σ* MDL was 0.25 pptv for a 10 s acquisition time, which improved with further signal averaging to 0.09 pptv with a 400 s acquisition time. In 2016, an innovative improvement of the IBBCEAS technique for NO_3_ detection was reported by Langridge et al. [42]. In this paper, a SC radiation source was firstly used for IBBCEAS technique, which is suitable for its broad wavelength coverage and high spectral brightness. The SC source processes an emission wavelength range from 400 nm to 2000 nm. By using the optical filter, the effective operating bandwidth of this technique referring to the highly reflective bandwidth of mirrors was limited to ~100 nm. The high-finesse optical cavity was 1.15 m long formed by two mirrors (*R* ~ 99.995%), leading to a MDL for NO_3_ of 3 pptv in a 2 s acquisition time with a corresponding sensitivity of 2.4 × 10^−9^ cm^−1^·Hz^−1/2^ at a 3*σ* noise level. For example, Table 3 describes the NO_3_ detection performances of the gas sensors implemented with the IBBCEAS technique, including the wavelength range, the effective optical path length, the minimum detection limit as well as the dynamic range.

#### 4.3.2. NO_2_

The reported IBBCEAS set-ups for NO_2_ detection are in several spectral range, including 410–490 nm, 540–580 nm and 615–655 nm. For the spectral range of 410–490 nm. In 2006, Langridge et al. [58] developed a portable device for the in-situ measurement of atmospheric NO_2_. They used a blue LED centered at 455 nm and a 1.5 m-long high stable optical cavity. Quantitative amounts of ambient NO_2_ (between 3 ppbv and 32 ppbv) were retrieved in the presence of ambient aerosol with a statistical uncertainty reaching 100 pptv for a 60 s averaging period. In 2008, Wu et al. [59] demonstrated a set-up based on a 457 nm-LED with a 92.5 cm-long cavity for NO_2_ detection. The measured NO_2_ concentration levels ranged from 39.54 ppbv (ng·mL^−1^) to 53.03 ppbv (ng·mL^−1^), with an uncertainty ranging from 3.1 ppbv (ng·mL^−1^) to 4.7 ppbv (ng·mL^−1^) during a sampling period of 80 s. In 2009, Wu et al. [60] introduced Allan variance plot into IBBCEAS to optimize averaging time for SNR enhancement. A MDL of ~2.2 ppbv (1*σ*) for NO_2_ was achieved at an optimal averaging time of 100 s. In 2011, a three channel-based IBBCEAS set-up was reported by Kennedy et al. [52]. The third channel is used for NO_2_ detection using a blue LED centered at 460 nm. The Allan deviation indicated a MDL of 5 pptv for NO_2_ in an averaging time of 1748 s at a pressure of 1 bar. In 2013, Ling et al. [61] used a blue LED centered at 462 nm with a 70 cm-long optical cavity (*R* ~ 99.9%). Atmospheric NO_2_ concentration levels (1 to 35 ppbv) during a seven-day period were retrieved from the absorption spectra between 444 nm and 468 nm. The results are in a good agreement (*R*^2^ = 0.975) with a commercial long path-differential optical absorption spectroscopy (LP-DOAS) device [88].

In order to avoid the effect of calibration errors of the mirror reflectivity on the quantitative result retrieved by traditional method, Ling et al. [45] presented a new retrieval method based on the measurement of atmospheric O_2_-O_2_ absorption. Experimental results showed that this method can be used to quantify multiple gas species using IBBCEAS, with the result insensitive to the calibration error of mirror reflectivity. In 2017, Liang et al. [62] presented an IBBCEAS set-up on the airplane to observe the actual atmospheric NO_2_ spatial distribution. The optical cavity fabricated by the Perfluoroalkoxy alkane (PFA) material for reducing wall losses, was formed by two high-reflectivity mirrors (*R* > 0.9999 @440–450 nm) separated by a distance of 1 m. The IBBCEAS system was deployed on an airborne platform, leading to a NO_2_ MDL of 95 pptv (1*σ*) with a time resolution of 2 s.

For the spectral range of 540–580 nm, in 2004, Ball et al. [34] demonstrated the first use of a green LED (535 nm) in IBBCEAS. The green LED light was coupled into a 1.5 m-long cavity. This set-up allows for a real time measurement of NO_2_ concentration levels of 64.8 ± 0.7 ppbv in an optimal acquisition time of 500 s. With respect to the spectral range of 615–655 nm, in 2008, Triki et al. [57] demonstrated a set-up based on a 643 nm-LED for NO_2_ detection. A 50 cm-long cavity was formed by two high-reflectivity mirrors (*R* ~ 99.92%). A MDL of <10 ppbv was communicated in a 1 s averaging time.

#### 4.3.3. HONO

For HONO measurement, the reported IBBCEAS device is in a spectral range of 360–375 nm using UV LED. In 2017, Nakashima et al. [63] reported an IBBCEAS set-up based on a UV-LED for HONO detection, with a 50 cm-long cavity formed by two mirrors (*R*~99.985%). A MDL of 0.2 ppbv for HONO was achieved in a 5 min acquisition time. In addition, good agreement (*R*^2^ = 0.94) was found between IBBCEAS and a commercial analyzer for the measurement of HONO.

#### 4.3.4. I_2_

In 2004, Ball et al. [34] developed an IBBCEAS instrument for the in situ measurement of atmospheric I_2_ using a green LED centered at 535 nm. Quantitative concentration levels of I_2_ of 991 ± 11 pptv were retrieved in a fitting range between 530 nm and 565 nm, and the RMS value of the residual spectra was 5.2 × 10^−9^ cm^−1^ for a 300 s averaging period. In 2014, Johansson et al. [64] demonstrated the application of an IBBCEAS to the detection of I_2_ at ambient pressure. The acquisition range was in the green spectral region around 500–550 nm using two mirrors with a reflectivity of ~99.99% and a distance of 0.5 m. A MDL of 0.04 nmol/L (~1 ppbv) without aerosols and 0.4 nmol/L (~10 ppbv) in the presence of aerosols were communicated.

#### 4.3.5. Industrial Pollutants Measurement (C_4_H_6_, C_4_H_8_O_2_)

1,3-butadiene (C_4_H_6_) is an industrially produced synthetic rubber and plastics and a harmful air pollutant. Furthermore, C_4_H_6_ can cause breathing problems and is a human carcinogen. Denzer et al. [35] developed an instrument by employing a near-IR SLED. Two types of detection schemes, including a dispersive monochromator with a lock-in and a Fourier transform interferometer, were demonstrated and compared with each other. The minimum detectable absorption coefficient were obtained to be 6.1 × 10^−8^ cm^−1^ using the dispersive spectrometer with the help of phase-sensitive detection and 1.5 × 10^−8^ cm^−1^ using the Fourier transform interferometer.

1,4-Dioxane (C_4_H_8_O_2_), one of the significant cyclic ethers with trace levels in the troposphere, are widely used in the field of biological, chemistry and textile. C_4_H_8_O_2_ is not biodegradable and was classified as a potential carcinogen by the US Environmental Protection Agency (EPA). Widely used as an industrial solvent, C_4_H_8_O_2_ is considered as the major anthropogenic source of 1,4-dioxane in the atmosphere [89,90]. Chandran et al. [65] reported a novel FT-IBBCEAS to record the absorption of C_4_H_8_O_2_ in the spectral range of 5900−8230 cm^−1^ with a resolution of 0.08 cm^−1^. Light from a SC source was coupled into a 644 cm-long optical cavity, leading to a MDL represented by an absorption coefficient of 8 × 10^−8^ cm^−1^ (~200 pptv) with an averaging time of 120 min.

#### 4.3.6. CO_2_, CH_4_, C_2_H_2_ Measurement

Almost all molecules exhibit strong absorption in the IR region due to fundamental vibrational-rotational transitions with specific molecular markers, which are capable of sensitive detection of many important atmospheric IR trace gas species using absorption spectroscopy [33]. In 2011, Denzer et al. [36] combined a SC source and a SLED with FT-IBBCEAS for spectroscopic and kinetic study of C_2_H_2_ and CO_2_. The established optical cavity consisted of two mirrors with a reflectivity of ~99.98% and a separation distance of 25 cm. Compared to SLED, a high detection sensitivity was achieved using the SC source, with a minimum detectable absorption coefficient of ~4 × 10^−9^ cm^−1^ at an acquisition time of 4 min.

In 2015, another innovative improvement of IBBCEAS technique, was reported by Aalto et al. [91], which allowed the sensing of CO_2_ and CH_4_ in the 1590–1700 nm. A tailored SC source was used with a 1.17 m-long cavity consisting of two mirrors (*R*~99.99%). A SLED was used in their set-up in order to perform a systematic comparison between the performance of a SC source and that of a SLED. A minimum detectable absorption coefficient (3*σ*) of 2.2 × 10^−9^ cm^−1^ and 6.2 × 10^−9^ cm^−1^ were achieved with the use of a SC and SLED source, respectively. In 2017, Amiot et al. [92], for the first time, presented the use of all-fiber SC source combined with IBBCEAS in the mid-IR wavelength range from 3000 nm to 3450 nm. A 1 m-long high-finesse optical cavity was formed by two mirrors (*R* ~ 99.95%), and good agreement was found between the measured and modelled absorption using the nominal concentrations with a sub parts-per-million in volume (ppmv) accuracy for CH_4_ and C_2_H_2_ detection, proving the feasibility of this sensing technique.

#### 4.3.7. H_2_O Measurement

The IBBCEAS technique also found applications in the field of spectroscopic investigation in a high temperature environment. In 2009, Watt et al. [93] demonstrated the combination of IBBCEAS with SC radiation for the measurement of the flame-generated H_2_O in the 1500 nm to 1550 nm region. Weak absorption lines of H_2_O at high temperature were observed in an acquisition time of ~30 min, which was hard to observe in a direct absorption measurement. The results demonstrated the efficiency of IBBCEAS for spectroscopic research in electrical and plasma discharges, or in flames at high temperature under a steady state.

Since the strong, narrow absorption lines of H_2_O are under-resolved and hence exhibit non-Beer–Lambert law behavior at the resolution encountered in IBBCEAS experiments, a new treatment was proposed by Langridge et al. [56], by calculating the effective absorption cross sections for fitting the differential structure in IBBCEAS spectra. The ring-down time of the cavity, the concentration of the absorber and the detailed spectroscopy of the absorber over the narrow range of wavelengths sampled onto the CCD detector pixel are all considered in the calculation of the effective absorption cross sections. This approach was tested on the IBBCEAS spectra of water vapor’s 4*υ* + *δ* absorption bands around 650 nm, and the retrieved water amounts agreed well with the measurement results of the relative humidity from a commercial hygrometer. Furthermore, Aalto et al. [91] reported a DOAS-type fitting method for data analysis that properly account for the combined effect of absorption line saturation and limited spectral resolution. In this model, the single-pass gas absorption was given by a linear approximation of Beer-Lambert law and the measured transmittance in the presence of a gas sample was convolved with the instrument slit function since it directly affected the transmittance spectrum. In contrast to previous approaches, this method is able to cope with a wide range of strong and weak absorption features typically of multi-component measurements, leading to a greatly enhanced dynamic range.

## 5. Target Gas Species in Field Measurement

In order to validate the performance of the developed IBBCEAS instrument, a large variety of measurements were performed under ambient conditions. High-power LEDs are compact, power efficient, inexpensive, stable in photon fluence and have a long life (>10,000 h), which makes them suitable for an IBBCEAS system in field measurements. As shown in Table 4, most of the target gas species in field IBBCEAS measurements are nitrogen oxides and nitrous acid (NO_2_, NO_3_, N_2_O_5_, HONO). Several platforms are demonstrated for field measurements, including marine boundary layer, aircraft, urban environment and suburban site. The measurement time varied from 1 h to 21 days, resulting in different concentration levels between several pptv and tens of ppbv. Temporal resolution is defined as the acquisition time of a single spectra of the target gas [54,62]. The measured concentration range refers to the concentration level ranges observed during the field measurements where the reported sensors were used. In order to verify the accuracy of the results acquired by the IBBCEAS set-ups, their performance was also compared with the commercial analytical instruments.

### 5.1. Marine Boundary Layer Measurement

For better understanding the atmospheric chemistry of reactive trace gases such as NO_3_ and N_2_O_5_ in the marine boundary layer, Langridge et al. [56] reported the performance of a red LED-based IBBCEAS system for field operation in the marine boundary layer in summer 2006. In this experiment, the mixture of NO_3_ and N_2_O_5_ made during the RHaMBLe field campaign was measured at Roscoff on the northern coast of Brittany, France. The IBBCEAS instrument was housed in a shipping container on the quayside in front of the station. NO_3_ is produced in the atmosphere by the reaction between NO_2_ and O_3_ and is photolyzed in sunlight. The concentration of NO_3_ and N_2_O_5_ was found to increase after sunset and decrease again at night.

### 5.2. Airborne Measurement

In order to obtain the concentration variation of the gas species in the tropospheric atmosphere, airborne experiments have been carried. In 2011, Kennedy et al. [52], for the first time, developed an IBBCEAS instrument for airborne usage with three separate optical channels. This instrument offers a capability of in-situ measurement of NO_3_, N_2_O_5_ and NO_2_ in the “RONOCO” campaign. During the flight, concentration levels of NO_3_ and N_2_O_5_ changed from below 2 pptv up to ~200 pptv and 600 pptv, respectively. Concentration levels of NO_2_ varied from below 50 pptv up to 12 ppbv in the polluted zones. Compared to the IBBCEAS data in this flight with a photolytic chemiluminescence-based detector, a strong correlation (*R*^2^ = 0.99) was achieved, demonstrating the high reliability of the IBBCEAS instrument in airborne deployment. In 2016, Liang et al. [62] measured NO_2_ in Anhui, China. The NO_2_ concentration value varied from 4 ppbv to 31 ppbv with an average value of 11.98 ppbv. Furthermore, they carried out an airborne NO_2_ measurement in Hebei province, China. The concentration levels were ~10.6 ppbv on the ground, and dropped sharply to ~2 ppbv at an altitude of 700 m. Subsequently, the NO_2_ concentration decreased to below the MDL of 95 pptv with the further increment of flight altitude. By using the airborne observation, the profile informatio of NO_2_ over the tropospheric atmosphere of Shijiazhuang and other areas in North China was obtained.

### 5.3. Urban Environment Measurement

Due to the active human activities and environmental pollution, ambient gas observations at urban sites are very important. With a blue LED-based IBBCEAS, Langridge et al. [58] measured the NO_2_ concentration levels in ambient air in October 2006. During the 38 h’ measurement, NO_2_ concentration levels varied from 3 ppbv to 34 ppbv, and compared with those obtained with a commercial chemiluminescence detector, a high correlation with an *R*^2^ coefficient of 0.998 was reported. In 2012, Ling et al. [61] measured atmospheric NO_2_ concentration levels with their IBBCEAS instrument. During seven-day’s measurements in May 2012, NO_2_ concentration levels varied from 1 ppbv to 35 ppbv. In order to verify the accuracy of the IBBCEAS set-up, a LP-DOAS device was used to measure atmospheric NO_2_. A correlation (*R*^2^) of 0.983 indicates the good agreement between the IBBCEAS and LP-DOAS device. In 2014, Duan et al. [55] recorded HONO and NO_2_ concentration levels for three days with their IBBCEAS instrument. The HONO concentration varied from below the MDL to 3.49 ppbv with an averaging value of 0.68 ppbv and the NO_2_ concentration ranged from 2.86 ppbv to 51.6 ppbv with an average value of 14 ppbv. In addition, the results obtained by the IBBCEAS set-up were compared with a DOAS set-up. The linear correlation coefficient of NO_2_ was 0.937 (*R*^2^ = 0.937), and the value of HONO was 0.917 (*R*^2^ = 0.917). The obtained correlation verified the accuracy of the IBBCEAS technique for simultaneous measurement of atmospheric HONO and NO_2_ concentration levels.

In 2016, Wang et al. [44] successfully carried out two comprehensive field campaigns in the winter and summer in Beijing. The used instrument was capable of measuring NO_3_ and N_2_O_5_ in one channel simultaneously. In these two campaigns, up to 1 ppbv of NO_3_ and N_2_O_5_ was observed with the presence of high aerosol compositions. In the summer campaign, up to 50 pptv of NO_3_ was present at night, which indicates active chemistry at night in Beijing. In the same year, Nakashima et al. [94] recorded numerous trace gas species in winter in Tokyo including SO_2_, O_3_, NO, NO_2_ and HONO. The HONO concentration levels ranged from less than the MDL of 0.2 ppbv to 7.1 ppbv, which changed similarly to the NO_2_ and NO concentrations. At the same time, the ratio of HONO to NO_2_ was also recorded. The variation trend was the same as that of HONO, with a range of the ratio from 0.002 to 0.19. Moreover, during the 21-day measurement period, it was found that the concentration levels were higher during the night than during the day. This is because photolysis occurs during the day and the height of the boundary layer is lower during the winter night. In addition, the NO_2_ concentration levels measured by IBBCEAS agreed well (*R*^2^ = 0.94) with that obtained by the cavity attenuated phase-shift spectroscopy (CAPS)-NO_2_ analyzer [94].

### 5.4. Suburban Site Measurement

With respect to the trace gas sensing in the field campaign at a suburban site, for the first time, a UV LED-based IBBCEAS device was developed by Wu et al. [54] for simultaneous measurements of HONO and NO_2_ in 2012. Field intercomparison campaign at a suburban site of Tung Chung in Hong Kong was performed and daytime and nighttime concentration levels of HONO and NO_2_ were recorded. Atmospheric HONO concentration levels of up to ~2 ppbv were obtained during the observation period. And the observed HONO to NO_2_ ratios (varying between ~0 and 0.45) indicate a very complex nocturnal environment for HONO formation at Tung Chung. A correlation (*R*^2^ = 0.7) of HONO concentration between the IBBCEAS and the LOPAP device was achieved, together with the correlation of NO_2_ concentration measurements of *R*^2^ = 0.82 between the IBBCEAS and a commercial NO_2_ analyzer.

In 2017, Fang et al. [20] firstly reported the development of a cage-based IBBCEAS instrument for ambient measurements of NO_2_ and CHOCHO in China’s Pearl River Delta (PRD) and Yangtze River Delta (YRD) regions. During these measurements, concentration levels of NO_2_ and CHOCHO changed from 2 ppbv to 15 ppbv and below 28 pptv up to ~180 pptv, respectively. Moreover, the Kalman adaptive filtering technique was applied to IBBCEAS based measurement, which efficiently reduced the real-time noise and improved the retrieval accuracy without affecting the time resolution.

## 6. Outlook

The development of the IBBCEAS technique was significant in recent years, and further improvements of the IBBCEAS are expected, including a higher detection sensitivity, a wider spectral coverage, an improved spectral resolution and the potential for sensor system integration.

(1) Sensitivity. There are a number of improvements of the IBBCEAS technique that can lead to higher detection sensitivity. First, the optical throughput and the sensitivity can be improved by using new developed SC radiation sources, owing to their combination of high spectral brightness and broadband wavelength coverage [95,96]. The application of LED in IBBCEAS will become more extensive with the increase of the intensity and stability of LED. Second, the SNR can be improved by suppressing the optical feedback using an optical isolator or energy-efficient spectral filters. In addition, the minimum detection limit can be enhanced by employing high-finesse cavities as well as optimizing coupling parameters. Furthermore, the accuracy and sensitivity can also be improved by using high-order correction parameters in the calibration of the mirror reflectivity [91].

(2) Spectral coverage. In order to increase the spectral bandwidth, the spectral range of the mirror with high reflectivity should be expanded through further progress in optical fabrication technology. The use of uncoated reflective optics (such as prisms [97,98]) is an advancement which enables the use of cavities over a large spectral range. Furthermore, the use of SC radiation source is a recent advancement for access to a wide spectral range from the UV [99] to the mid-IR [100]. The supercontinuum radiation source which outperforms conventional thermal sources in terms of brightness and collimation is of particular interest for trace gas sensing where strong mid-IR absorption bands associated with fundamental vibrational transitions can be measured [91]. Further improvements of output powers and spectral flux stability of SC light sources enable the application of mid-IR absorption spectroscopy without the use of expensive mid-IR lasers (e.g., quantum cascade laser, interband cascade laser) [101].

(3) Spectral resolution. The spectral resolution could be improved in a variety of ways. By replacing a scanning spectrum analyzer with a detector array, the spectral acquisition time can be reduced, leading to a high temporal resolution and an increased sensitivity [93] In addition, this improvement will also decrease the sensor cost and make it more compact [91] Secondly, the FT-IBBCEAS can be used to record the absorption property of a gas molecular over a broad spectral region with sufficiently high resolution. This technique is helpful to identify weak absorption of specific gaseous species, together with many other useful applications, including spectral studies of isotopic samples, flames, plasma discharges or detection of chemical sources [65].

(4) System miniaturization and integration. Due to conceptual simplicity and robustness, the IBBCEAS technique is ideally suitable for trace gas sensing applications. The future of this technique will focus on developing portable instrumentation which is capable of multiple gas species detection with high sensitivity, wide bandwidth, optimal spectral resolution as well as high temporal resolution.

## 7. Conclusions

Due to experimental simplicity, high sensitivity and high temporal resolution, the IBBCEAS technique has been an important topic in the field of absorption spectroscopy. IBBCEAS can be described as the combination of CEAS and DOAS, leading to unique advantages of compactness and robustness with short integration time (seconds to minutes) as well as multiple gas species analysis. In this review, we addressed the facts about the principle and evolution of the IBBCEAS technique together with the achievement of the application of this technique in trace gas sensing. Due to a broad spectral range, the IBBCEAS technique is suitable for the proof-of-principle measurement of weak atmospheric transition and overtone bands of gas molecules, and also for the detection of many atmospheric trace gas species (e.g., NO_2_, NO_3_, HONO, CO_2_). Field measurements using IBBCEAS under ambient conditions were also discussed. The advances of IBBCEAS technique opens up new research paths for observing the spatial and long-term trends of atmospheric changes. The technique will further improve our vision and understanding of the tropospheric processes and trends, which will have a significant effect on predicting the atmospheric changes in the future.

## Figures and Tables

**Table 1:** Reported light source, cavity and detection scheme used in IBBCEAS.

Items	Classification	Reference	Advantages	Disadvantages
Light Source	Short-arc Xe lamp	Fiedler et al. (2003)	high spectral brightness;	intensity fluctuations;
		Ashu-Ayem et al. (2012)	broad spectral region;	high energy consumption;
	LED	Wu et al. (2009)	high brightness, low power consumption;	sensitive to temperature and current fluctuations;
		Wang et al. (2017)	narrow full width at half maximum (FWHM);	
	SC source	Chandran et al. (2016)	high power density and broadband wavelength coverage;	unstable in a long acquisition periods and costly
Cavity Scheme	Closed-path	Gherman et al. (2008)	enabling the design of a portable system with good mechanical stability and compactness;	wall losses for different target species;
		Amiot et al. (2017)		
	Open-path	Varma et al. (2009)	simple calibration procedure;	at a high aerosol concentration levels,
		Ling et al. (2013)	free from wall loss of target species;	the concentration retrieval is challenging;
		Nakashim et al. (2017)	arbitrarily extended cavity length;	a reference spectrum *I*_0_ is not available;
Detection Scheme	dispersive approaches	Kennedy et al. (2011)	rapid detection over a wide wavelength range with a multi-channel detector;	low spectral resolution of ~0.1 nm and ~1 nm;
		Liang et al. (2017)		
	interferometric approaches	Orphal et al. (2008)	high resolution and sensitivity of ~0.02 cm^−1^ and ~4 cm^−1^ from VIS to near-IR range;	long acquisition time;
		Denzer et al. (2011)		not compact and costly;

**Table 2:** Performances of the IBBCEAS instrument for target gas species in laboratory.

Species Measured	Light Source	Reflectivity (%) (Radius of Curvature)	Cavity Length (m)	Spectral Resolution (nm)	Minimum Detection Limit (Acquiring Time)	Retrieval Range (nm)	Ref.
NO_3_, NO_2_	Xe lamp	99.99 (5 m)	4.62	0.3	NO_3_ (4 pptv) NO_2_ (10 ppbv) (57 s)	645–675	[25]
	Xe lamp	99.9 (21 m)	20	0.6	NO_3_ (2 pptv) NO_2_ (2 pptv) (5 s)	630–690	[9]
	Red-LED	99.98 (1 m)	0.5	2.3	NO_3_ (2 pptv) NO_2_ (600 pptv) (400 s)	610–640	[50]
	Red-LED	99.98 (2 m)	2	0.77	NO_3_ (7.9 pptv) NO_2_ (9 ppbv) (60 s)	638–672	[51]
NO_3_, N_2_O_5_	Red-LED	99.99 (6 m)	0.94	0.9	NO_3_ (1.1 pptv) N_2_O_5_ (2.4 pptv) (850 s)	615–706	[52]
	Red-LED	99.99 (1 m)	0.5	0.85	NO_3_ (2.4 pptv) N_2_O_5_ (2.7 pptv) (1 s)	640–680	[44]
HONO, NO_2_	UV-LED	99.85 (not available)	1.15	0.35	HONO (~4 ppbv) NO_2_ (~14 ppbv) (20 s)	360–380	[53]
			4.5	0.5	HONO (~0.13 ppbv) NO_2_ (~0.38 ppbv) (10 min)	360–380	
	UV-LED	99.97 (2 m)	1.85	0.77	HONO (~430 pptv) NO_2_ (~1 ppbv) (90 s)	358–378	[47]
	UV-LED	99.9 (2 m)	1.76	0.53	HONO (~0.3 ppbv) NO_2_ (~1 ppbv) (120 s)	353–376	[54]
	UV-LED	99.99 (not available)	0.55	0.5	HONO (~0.22 ppbv) NO_2_ (~0.45 ppbv) (320 s)	359–387	[55]
CHOCHO, NO_2_	Xe lamp	99.9966 (1 m)	0.944	0.54	CHOCHO (29 pptv) NO_2_ (30 pptv) (60 s)	404–532	[27]
	Blue-LED	99.98 (not available)	0.42	0.35	CHOCHO (8 pptv) NO_2_ (40 pptv) (21 s)	440–480	[20]
I_2_, IO, OIO	Xe lamp	99.99 (2m)	1.45	0.4	I_2_ (26 pptv,60 s) OIO (45 pptv, 5 s)	525–555	[26]
		99.98 (10m)		0.2	IO (210 pptv, 60 s)	420–460	
	Xe lamp	99.97 (not available)	1.99	0.96	I_2_ (not available) OIO (not available)	520–560	[32]
					IO (not available)	420–460	
NO_3_	Red-LED	99.995 (6 m)	1.9	0.45	NO_3_ (2.5 pptv) (516 s)	652–672	[34]
	Red-LED	99.99 (1 m)	1.1	0.38	NO_3_ (0.25 pptv)	651–672	[56]
	SC	99.995 (1 m)	1.15	0.3	NO_3_ (3 pptv) (3*σ*)	640–675	[42]
NO_2_	Green-LED	99.99 (6 m)	1.5	0.09	NO_2_ (64.8 ± 0.7 ppbv) (500 s)	540–580	[34]
	Red-LED	99.92 (0.5 m)	0.5	1.85	NO_2_ (<10 ppbv) (1 s)	615–655	[57]
	Blue-LED	99.976 (6 m)	1.5	0.33	NO_2_ (100 pptv) (60 s)	441–462	[58]
	Blue-LED	99.7 (1 m)	0.925	0.89	NO_2_ (3.1–4.7 ppbv) (60 s)	472–480	[59]
	Blue-LED	99.7 (1 m)	0.975	0.9	NO_2_ (2.2 ppbv) (100 s)	450–490	[60]
	Blue-LED	99.9 (1.5 m)	0.7	0.3	NO_2_ (9.6 ppbv) (90 s)	444–468	[61]
	Blue-LED	99.985 (6 m)	1	0.5	NO_2_ (95 pptv) (2 s)	450–470	[62]
	Blue-LED	99.99 (6 m)	0.94	0.4	NO_2_ (5 pptv) (1748 s)	410–482	[52]
HONO	UV-LED	99.985 (not available)	1	0.5	HONO (~0.2 ppbv) (5 min)	360–375	[63]
I_2_	Green-LED	99.99 (6 m)	1.5	0.09	I_2_ (991 ± 11 pptv) (300 s)	530–565	[34]
	Green-LED	99.99 (1 m)	0.5	0.5	I_2_ (0.04 nmol/L, ~1 ppbv)	500–550	[64]
C_4_H_6_	SLED	99.98 (not available)	0.25	1.8 cm^−1^	dispersive spectrometer (6.1 × 10^−8^ cm^−1^)	1620–1700	[35]
				0.5 cm^−1^	FTS (1.5 × 10^−8^ cm^−1^)		
C_4_H_8_O_2_	SC	99.9 (6 m)	6.44	0.08 cm^−1^	8 × 10^−8^ cm^−1^ (~200 pptv, 120 min)	1215–1700	[65]

**Table 3:** Performances of the IBBCEAS instrument for NO_3_ detection.

Wavelength Range (nm)	Effective Optical Path Length (m)	Minimum Limit Detection (pptv)	Dynamic Range (pptv)	Ref.
628–688	2000	4	Not stated	[25]
630–690	2000	2	Not stated	[9]
610–640	25,000	2	Not stated	[50]
638–672	22,000	7.9	0–185	[51]
645–680	9400	1.1	Not stated	[52]
640–680	5000	2.4	0–64	[44]
652–672	38,000	2.5	0–43	[34]
651–672	11,000	0.25	Not stated	[56]
640–675	19,200	1	0–38	[42]

**Table 4:** Performances of the reported IBBCEAS for target gas species in field measurements. * the correlation between IBBCEAS set-up and the commercially analytical instrument.

Different Platforms	Light Source	Gas	Location	Date	Duration	Average Concentration	Measured Concentration Range	Minimum Detection Limit	Temporal Resolution	*R*^2^ *	Ref.
Marine boundary layer	Red-LED	NO_2_, NO_3_, N_2_O_5_	Brittany, France	September 2006	15 h	Not stated	NO_3_ (above 1 ppbv)	NO_3_ (0.25 pptv)	10 s	Not stated	[56]
Aircraft	Red-LED	NO_2_, NO_3_, N_2_O_5_	North Sea and Thames Estuary, UK	July 2010	5 h	Not stated	NO_3_ (<2–200 pptv) N_2_O_5_ (<2–600 pptv) NO_2_ (<0.05–12 ppbv)	NO_3_ (1.1pptv) N_2_O_5_ (2.4 pptv) NO_2_ (5 pptv)	NO_3_ (1.2 s) N_2_O_5_ (8 s) NO_2_ (1 s)	0.99	[52]
	Blue-LED	NO_2_	Anhui, China	March 2016	36 h	~11.98 ppbv	4–31 ppbv	NO_2_ (95 pptv)	2 s	0.86	[62]
Urban site	Red-LED	NO_3_, N_2_O_5_	Beijing, China	February, May 2016	7 days	Not stated	NO_3_ + N_2_O_5_ (1 ppbv) NO_3_ (up to 50 pptv)	NO_3_ (2.4 pptv) N_2_O_5_ (2.7 pptv)	1 s	Not stated	[44]
	Blue-LED	NO_2_	Cambridge, UK	October 2006	38 h	Not stated	3–34 ppbv	NO_2_ (100 pptv)	60 s	0.9982	[58]
	Blue-LED	NO_2_	Anhui, China	May 2012	7 days	Not stated	1–35 ppbv	NO_2_ (9.6 ppbv)	90 s	0.983	[61]
	UV-LED	HONO, NO_2_	Anhui, China	March 2014	3 days	HONO (0.68 ppbv) NO_2_ (14 ppbv)	HONO (<0.22–3.49 ppbv) NO_2_ (2.86–51.6 ppbv)	HONO (0.22 ppbv) NO_2_ (0.45 ppbv)	20 min	HONO (0.917) NO_2_ (0.937)	[55]
	UV-LED	HONO	Tokyo, Japan	January 2016	21days	1.5 ± 1.1 ppbv	0.2–7.1 ppbv	HONO (0.2 ppbv)	20 min	0.94	[63]
Suburban site	UV-LED	HONO, NO_2_	Tung Chung, Hong Kong	May 2012	2 days	Not stated	HONO (up to~2 ppbv)	HONO (0.3 ppbv)	2 min	HONO (0.7)	[54]
	Blue-LED	NO_2_, CHOCHO	PRD and YRD regions, China	August 2017	3 days	Not stated	NO_2_ (2–15 ppbv) CHOCHO (<28–180 pptv)	NO_2_ (40 pptv) CHOCHO (8 pptv)	21 s	Not stated	[20]
